# Case Report: Immune-mediated acute liver failure induced by tislelizumab in a patient with advanced cervical cancer

**DOI:** 10.3389/fonc.2025.1604601

**Published:** 2025-08-22

**Authors:** Chaoxia Liu, Xinchun Li, Yuping Deng

**Affiliations:** Hunan Cancer Hospital, The Affiliated Cancer Hospital, Xiangya School of Medicine, Central South University, Changsha, Hunan, China

**Keywords:** tislelizumab, immune-mediated hepatitis, acute liver failure, immune checkpoint inhibitor toxicity, cervical cancer

## Abstract

Tislelizumab, an anti-PD-1 monoclonal antibody, is associated with immune-related hepatitis in 1.8% of cases, but reports of acute liver failure (ALF) remain exceedingly rare. We present a case of fulminant hepatitis and ALF following Tislelizumab therapy in a 55-year-old woman with locally advanced cervical adenocarcinoma. After three cycles of concurrent chemoradiotherapy and Tislelizumab, she developed grade 4 immune-mediated hepatitis and ALF following a fourth Tislelizumab dose, marked by severe transaminitis (AST 5329 U/L, ALT 2384 U/L), coagulopathy (INR 5.85), hyperbilirubinemia (TBIL 56.99 IU/L), and hepatic encephalopathy. Management included plasma exchange, continuous hemofiltration, high-dose corticosteroids, and immunosuppressive agents. Despite aggressive intervention, the patient’s condition deteriorated, underscoring the rapid progression of Tislelizumab-induced hepatotoxicity. This case highlights the critical need for vigilant monitoring of high-risk patients receiving immune checkpoint inhibitors and early intervention for suspected immune-mediated liver injury.

## 1 Introduction

Tislelizumab, an anti-human programmed death receptor-1 (PD-1) monoclonal IgG4 antibody, has been approved for clinical application in several cancers and advanced solid tumors. Ever since the approval of Tislelizumab, the incidence of immune-related hepatitis found in clinical practice is relatively low at 1.8% (1, 2). There’s, however, rare reports on acute liver failure induced by this drug. Herein, we are reporting a rare case of fulminant hepatitis in company with acute liver failure following the treatment with Tislelizumab. This case highlights the potential for severe adverse events associated with hepatic dysfunction under the use of immunotherapy agent, and the close monitoring for those at high risk is urgently needed.

## 2 Case presentation

On September 19th, 2024, a 55 year old female was admitted to our hospital with fatigue, anorexia, and abdominal discomfort for 5 days. Clinically, she was diagnosed with locally advanced cervical cancer (stage IIIC2r cervical adenocarcinoma). There were no evidence of metastases, nor was there any history of hepatitis or hepatic damage. The result of PD-L1 test was positive (CPS 5). Subsequently, from June 27th to August 26th, 2024, this patient underwent concurrent chemoradiotherapy (the dose of 95% planning target volume was 46.8 Gy by 26 times, the dose of lymph node was 58.8 Gy by 26 times, accompanied with Cisplatin on the dose of 40mg/m^2^ by 4 times, the dose of intracavitary brachytherapy volume is 6 Gy by 5 times), and received Tislelizumab treatment three times. During this period, she experienced a decline in platelet counts at 27×10^9^/L. There was, however, no evidence of development of immune-mediated hepatotoxicity or other severe adverse reactions. After her results of blood routine test (blood RT) returned to normal, she received the fourth course of maintenance monotherapy of Tislelizumab on September 14th, 2024.

**Figure 1:**
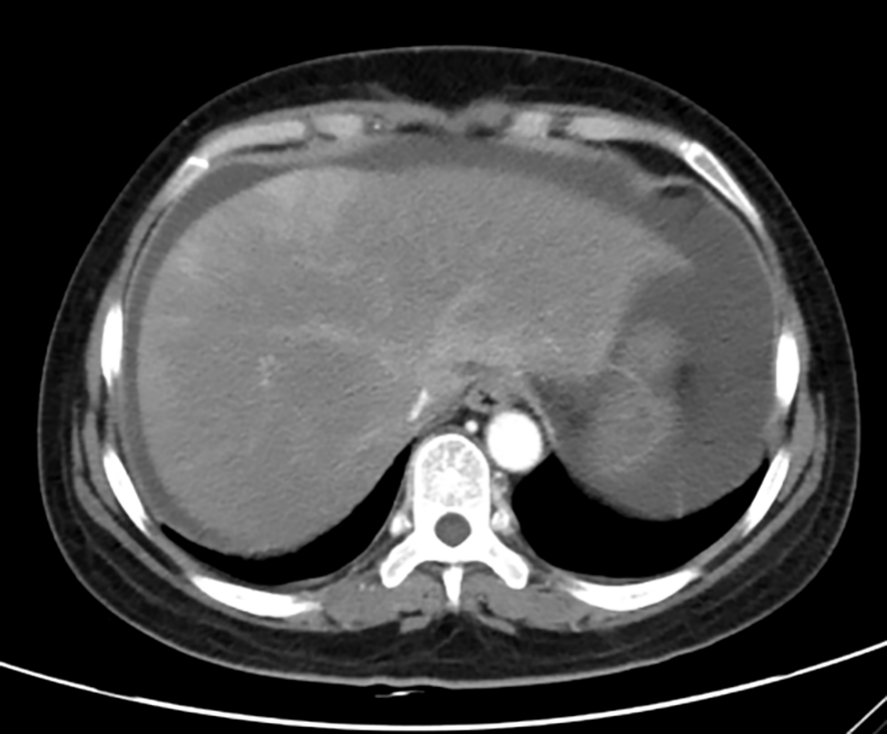
Enhanced CT scan of liver (Decreased and uneven density of liver parenchyma, increased abdominal and pelvic effusion, thus, drug-induced liver injury was considered).

Unfortunately, two days later, her symptoms deteriorated. On examination, she had abdominal distention and positive mobility dullness, but her temperature was normal. She was conscious, and had no icteric sclera, and no obvious ecchymosis on the skin. Those results of blood RT on September 19th were as follow: white blood cells count at 11.75×10^9^/L, hemoglobin at 102 g/L, platelet at 10×10^9^/L. Comprehensive metabolic panel (CMP) showed: aspartate aminotransferase at 3980 U/L (≦ 40 IU/L), alanine aminotransferase at 1670 U/L (≦ 35 IU/L), creatinine at 117.2 ummol/L (≦ 90 ummol/L), uric acid at 742.3 ummol/L (≦ 357 ummol/L), urea at 14.5 ummol/L (≦ 7.5 ummol/L), Na+ at 128 mmol/l (≧ 137 mmol/L), prothrombin time (PT) at 42.30 sec, international normalized ratio (INR) at 3.96 sec, activated partial thrombin time at (APTT) 34.20 sec, thrombin time (TT) at 25.30 sec, fibrinogen (FIB) concentration at 0.71 g/L. The test of hepatitis A, B, C, E serologies was negative. T-lymphocyte subsets were normal, but interleukin-6 was abnormally elevated to 29.5 pg/ml (≦ 7 pg/ml). Accordingly, she was diagnosed with immune-mediated hepatitis G4 and acute liver failure. Immediately, the patient was transferred to intensive care unit (ICU) department at 8:00 PM on September 19th. At 10:00 PM, she went into delirium, and obvious ecchymosis appeared on her skin. The re-examined CMP results were as follow: Aspartate transaminase at 5329 U/L, alanine transaminase at 2384 U/L, total bilirubin (TBIL) at 56.99 IU/L (≦ 23 IU/L), direct bilirubin (DBIL) at 30.23 IU/L (≦ 4 IU/L), albumin at 32 g/L, PT at 62.50 sec, INR at 5.85 sec, APTT at 41.40 sec, TT at 27.80 sec, FIB concentration at 0.5 g/L, Hb at 83 g/L, platelet count at 12×10^9^/L, blood ammonia at 210 IU/L (≦ 72 IU/L). The results of blood gas in emergency were as follow: PH at 7.362, PCO2 at 19.5 mmHg, PO2 at 155 mmHg, HCO3- at 14.4 mmol/L, BE- at 14.3 mmol/L, Na+ at 125mmol/L, Lactic acid at 15 mmol/L, glucose at 8.3 mmol/L. Computer tomography scan revealed that there were massive abdominal and pelvic effusion, and suspected drug-induced liver injury (**Figure 1**). A liver biopsy was not performed for the sake of poor coagulation and low platelets.

Upon admission to ICU, the patient was receiving a comprehensive treatment plan, including artificial liver therapy in the form of plasma exchange and continuous hemofiltration. Additionally, she was prescribed prednisolone 160 mg every 24 hours intravenously and intravenous immunoglobulin 20 g daily for immune modulation. After 3 days, mycophenolate mofetil was added for immunosuppressive therapy. To prevent gastric bleeding, lansoprazole was administered. She also received calcium supplements, energy mixtures containing glutathione, glutathione, and magnesium oxalate for liver protection. Piperacillin tazobactam was used for infection prevention. When her Hb and platelet counts decreased, she received the infusion of red blood cell suspensions and platelet. Plasma and cryoprecipitate were infused daily to supplement blood coagulation factors, along with daily infusions of human blood albumin.

**Figure 2:**
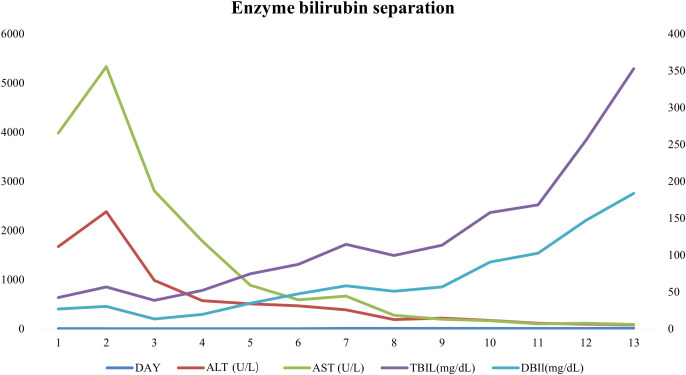
Changes of comprehensive metabolic panel results.

After 1 week, she awoke with decreased blood ammonia levels at 80 IU/L, and a gradual decrease in aspartate aminotransferase and alanine aminotransferase. Her bilirubin levels, however, increased gradually. Caspofungin was prescribed due to fungal infection. An abdominal ultrasonography revealed that cholestasis of the gallbladder, and significant ascites. After 10 days, her jaundice worsened with TBIL level increasing to 352 IU/L and DBIL to 187 IU/L (**Figure 2**). Her platelet count dropped to 15×10^9^/L Despite the use of a double plasma molecular adsorption system, its effect was not limited, and she had lung inflammation and heart failure. Ultimately, she succumbed to multiple organ dysfunction syndrome on October 1st, 2024.

## 3 Discussion

During monotherapy with immune checkpoint inhibitors (ICIs), the immune-mediated liver injury caused by ICls (ILICI) occurs in 5%~10% of all patients (2), with 1%~2% experiencing the severity of grade 3. Nevertheless, It’s reported that fulminant hepatitis accounts for less than 0.5% of all treated patients (1, 3). Here, we presented a case of immune-mediated liver failure induced by Tislelizumab. The patient’s condition rapidly deteriorated, leading to the prompt onset of hepatic encephalopathy. Given the primary elevation of her transaminases, we suspected a hepatocellular pattern of drug-induced liver injury. Immune-mediated hepatotoxicity (IMH) is a clinical diagnosis of exclusion. The role and timing of liver biopsy in IMH are still debated (1). Liver biopsies are not always necessary to diagnose IMH in a patient presenting with typical features after ICIs exposure, who have thorough serological and imaging investigations to exclude alternative etiologies and who respond to the initiation of steroid therapy with down trending ALT within 5 to 7days (2). The patient in this case was primarily diagnosed according to the above - mentioned criteria. Liver biopsy was not carried out owing to coagulation dysfunction and an extremely low platelet count. In accordance with the European Association for the Study of the Liver Clinical Practice Guidelines on the Management of Hepatic Encephalopathy (2022 Version) and the Chinese Guideline for Diagnosis and Management of Drug-induced Liver Injury (2023 Version) (4), the patient underwent plasma exchange and hemofiltration with an artificial liver. Additionally, she received high doses of immunosuppressive agents such as corticosteroids, intravenous immunoglobulin, and even mycophenolate mofetil. Unfortunately, her coagulation function failed to improve, and biliary enzyme separation occurred. When her TBil levels doubled to 352, a double plasma molecular adsorption system was implemented, effectively reducing bilirubin level. Dual-molecule plasma adsorption system (DPMAS) treatment with plasma exchange (PE) has a curative effect on ILICI because it can remove inflammatory factors, bilirubin, creatinine, and other small molecular substances from blood (1) However, despite these efforts, the patient ultimately succumbed to multiple organ dysfunction syndrome, including pulmonary fungal infection, heart failure, liver failure, coagulation disorders, and thrombocytopenia.

Potential risk factors for hepatotoxicity induced by ICIs include: autoimmunity epidemic liver disease, combination therapy with immune checkpoint inhibitors, genetic factors, different cancer types and so on (5–8). Previous studies suggests that the PD-L1/PD-1 pathway plays a crucial role in immune regulation within the liver, aiding in the maintenance of tolerance through the modulation of CD8 T-cell activation and apoptosis (9). During anti-PD-1/PD-L1 therapy, it becomes evident how the threshold for hepatic inflammation may be lowered, enabling local T-cell activation and uncontrolled cytotoxicity (9). In word, ILICI are based on uncontrolled immune system response against hepatocytes (3, 4). Whether there are relevant biomarkers to predict the occurrence of immune-related adverse events, Interleukin-6 (IL-6), a significant pro-inflammatory cytokine, plays a pivotal role in initiating cytokine release syndrome. Hailemichael (10) suggests that IL-6 blockade abrogates immunotherapy toxicity and promotes tumor immunity. Low neutrophil-to-lymphocyte ratio(NLR<3)in peripheral blood at baseline may be significantly associated with the development of irAEs (5). Potential biomarkers to predict ILICI in clinical practice are still needed. In this patient, we observed NLR=1.79 in peripheral blood at baseline but abnormally elevated levels of IL-6 at the time of liver injury. The prognosis of fulminant hepatitis is extremely poor, and it is of great significance to identify patients at high risk of severe immune side effects in advance.

## 4 Limitations

This study has several limitations. First, the lack of histopathological confirmation of immune-mediated liver injury due to the patient’s contraindications for liver biopsy (severe coagulopathy) limits definitive diagnostic certainty. Second, the single-case design inherently restricts generalizability, as individual variations in disease progression and treatment responses cannot be extrapolated to broader populations. Third, the potential interaction between chemoradiotherapy and Tislelizumab in triggering hepatotoxicity remains unclear. Although the interval between the patient’s last chemotherapy session (July 29, 2024) and the onset of liver injury (September 19, 2024) was seven weeks, during which only Tislelizumab was administered, and pelvic radiotherapy typically spares the liver, residual effects of prior therapies or synergistic toxicity cannot be entirely ruled out. Further studies are warranted to elucidate these uncertainties.

## Data Availability

The original contributions presented in the study are included in the article/supplementary material. Further inquiries can be directed to the corresponding author.
